# Lithium Treatment Improves Cardiac Dysfunction in Rats Deprived of Rapid Eye Movement Sleep

**DOI:** 10.3390/ijms231911226

**Published:** 2022-09-23

**Authors:** Pao-Huan Chen, Cheng-Chih Chung, Shuen-Hsin Liu, Yu-Hsun Kao, Yi-Jen Chen

**Affiliations:** 1 Graduate Institute of Clinical Medicine, College of Medicine, Taipei Medical University, Taipei 11031, Taiwan; 2 Department of Psychiatry, Taipei Medical University Hospital, Taipei 11031, Taiwan; 3 Department of Psychiatry, School of Medicine, College of Medicine, Taipei Medical University, Taipei 11031, Taiwan; 4 Division of Cardiovascular Medicine, Department of Internal Medicine, Wan Fang Hospital, Taipei Medical University, Taipei 11696, Taiwan; 5 Division of Cardiology, Department of Internal Medicine, School of Medicine, College of Medicine, Taipei Medical University, Taipei 11031, Taiwan; 6 Taipei Heart Institute, Taipei Medical University, Taipei 11031, Taiwan; 7 Division of Cardiology, Department of Internal Medicine, Shuang Ho Hospital, Taipei Medical University, New Taipei City 23561, Taiwan; 8 Department of Medical Education and Research, Wan Fang Hospital, Taipei Medical University, Taipei 11696, Taiwan

**Keywords:** rapid eye movement sleep deprivation, lithium, antifibrosis, cardioprotection

## Abstract

Rapid eye movement (REM) sleep deprivation triggers mania and induces cardiac fibrosis. Beyond neuroprotection, lithium has cardioprotective potential and antifibrotic activity. This study investigated whether lithium improved REM sleep deprivation-induced cardiac dysfunction and evaluated the potential mechanisms. Transthoracic echocardiography, histopathological analysis, and Western blot analysis were performed in control and REM sleep-deprived rats with or without lithium treatment (LiCl of 1 mmol/kg/day administered by oral gavage for 4 weeks) in vivo and in isolated ventricular preparations. The results revealed that REM sleep-deprived rats exhibited impaired contractility and greater fibrosis than control and lithium-treated REM sleep-deprived rats. Western blot analysis showed that REM sleep-deprived hearts had higher expression levels of transforming growth factor beta (TGF-β), phosphorylated Smad 2/3, and alpha-smooth muscle actin than lithium-treated REM sleep-deprived and control hearts. Moreover, lithium-treated REM sleep-deprived hearts had lower expression of angiotensin II type 1 receptor, phosphorylated nuclear factor kappa B p65, calcium release-activated calcium channel protein 1, transient receptor potential canonical (TRPC) 1, and TRPC3 than REM sleep-deprived hearts. The findings suggest that lithium attenuates REM sleep deprivation-induced cardiac fibrogenesis and dysfunction possibly through the downregulation of TGF-β, angiotensin II, and Ca^2+^ signaling.

## 1. Introduction

A decrease in an individual’s sleep duration is not only a core manifestation of mania but also a trigger for a manic episode [1,2]. Alterations in rapid eye movement (REM) density were observed in manic patients with reduced sleep [3]. Such changes in sleep architecture are recognized as a neurophysiological signature in individuals with bipolar disorder. In the laboratory, rats deprived of REM sleep have been well-established to induce manic-like behaviors over decades [4,5,6]. Moreover, rats deprived of REM sleep have been shown to aggravate profibrotic signaling [7,8], as well as numerous cardiac abnormalities, including myocardial hypertrophy, interstitial fibrosis, and pumping failure [8,9]. Activation of profibrotic signaling plays a role in the pathogenesis of heart failure in patients with bipolar disorder. Thus, studying lithium’s effect on profibrotic signaling in REM sleep-deprived hearts might have translational relevance to the clinical scenario [10,11].

Cardiac fibrosis is a detrimental factor associated with nearly all forms of heart disease [11]. In the process of cardiac fibrosis, the overactivation of cardiac fibroblasts results in the excessive deposition of extracellular matrix proteins, which in turn reduces tissue compliance, interferes with electrical conduction, and accelerates progression to heart failure [11,12]. Our recent study revealed that lithium at therapeutic doses reduced the migration and collagen synthesis ability of cardiac fibroblasts through its inhibitory effects on store-operated Ca^2+^ entry [13]. These findings are in line with those of other clinical studies that have suggested the cardioprotective potential of lithium [14,15]. Although heart failure is the primary cause of sudden cardiac death in patients with bipolar disorder [16], the role of lithium in REM sleep deprivation-induced cardiac dysfunction remains unclear. Thus, this experimental study examined the effects of lithium treatment on REM sleep deprivation-induced cardiac dysfunction in an animal model and evaluated the potential mechanisms. We hypothesized that lithium treatment may reduce cardiac dysfunction and fibrogenesis in REM sleep-deprived rats.

## 2. Results

### 2.1. Effect of Lithium Treatment on Cardiac Function in REM Sleep-Deprived Rats

**Figure 1:**
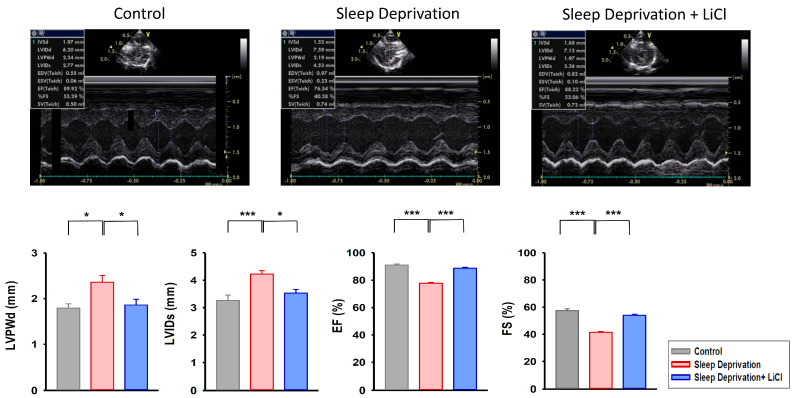
Echocardiographic study in REM sleep deprivation with and without lithium treatment. The REM sleep-deprived rats (*n* = 8) had increased left ventricular posterior wall thickness at end-diastole (LVPWd) and increased internal diameter at end-systole (LVIDs) compared with the control rats (*n* = 8) and lithium-treated REM sleep-deprived rats (*n* = 8). In addition, the REM sleep-deprived rats exhibited a lower left ventricular ejection fraction (EF) and lower fractional shortening (FS) compared with the control and lithium-treated REM sleep-deprived rats. * *p* < 0.05; *** *p* < 0.001.

As shown in Figure 1, the rats deprived of REM sleep had increased left ventricular posterior wall thickness at end-diastole (LVPWd) and internal diameter of the left ventricle at end-systole (LVIDs) compared with the control and lithium-treated REM sleep-deprived rats. In addition, the REM sleep-deprived rats exhibited lower ventricular ejection fraction (EF) and fractional shortening (FS) than did the control and lithium-treated REM sleep-deprived rats. On the other hand, the lithium-treated REM sleep-deprived rats had similar LVPWd, LVIDs, EF, and FS to the control rats.

### 2.2. Effect of Lithium Treatment on Cardiac Fibrosis in REM Sleep-Deprived Rats

**Figure 2:**
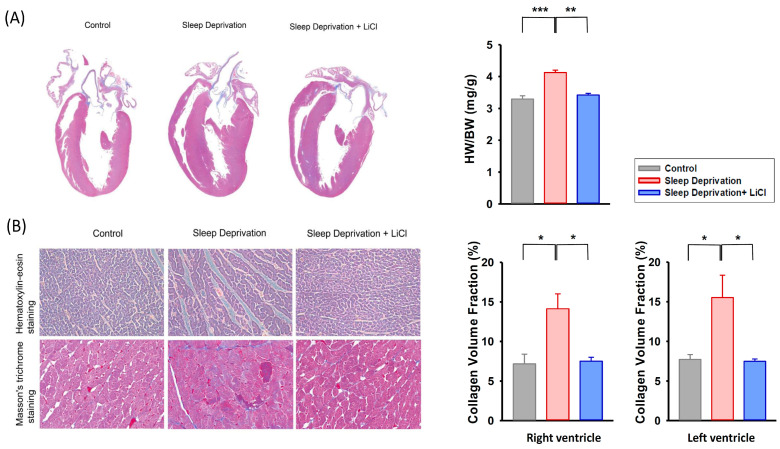
Cardiac morphology, histology, and fibrotic severity in REM sleep deprivation with and without lithium treatment. (**A**) Gross examination of a heart and its four-chamber view histology stained with Masson’s trichrome staining. The four-chamber view histology revealed that the REM sleep-deprived rats exhibited greater ventricular hypertrophy than did the control and lithium-treated REM sleep-deprived rats. The heart-to-body weight ratios were higher in the REM sleep-deprived rats (*n* = 3) than in the control (*n* = 3) and lithium-treated REM sleep-deprived (*n* = 3) rats. (**B**) The upper and lower panels show hematoxylin–eosin staining (original magnification, ×200) and Masson’s trichrome staining (original magnification, ×400) over the ventricles, respectively. The REM sleep-deprived rats (*n* = 3) exhibited a greater increase in fibrosis compared with the control (*n* = 3) and lithium-treated REM sleep-deprived (*n* = 3) rats. Fibrosis severity levels are expressed as collagen volume fractions. * *p* < 0.05; ** *p* < 0.01; *** *p* < 0.001.

Figure 2 illustrates the cardiac morphology and histology of the control and REM sleep-deprived rats with and without lithium treatment. The rats deprived of REM sleep exhibited more ventricular hypertrophy than did the control and lithium-treated REM sleep-deprived rats (Figure 2A). Furthermore, the heart-to-body weight ratios were higher in REM sleep-deprived rats than those of the control and lithium-treated REM sleep-deprived rats. Histological examination revealed that the REM sleep-deprived rat hearts exhibited more ventricular interstitial fibrosis than did the control rat hearts (Figure 2B). The lithium-treated REM sleep-deprived rat hearts exhibited less fibrosis than did the hearts of the REM sleep-deprived rats. Analysis of the collagen volume fraction revealed that the REM sleep-deprived rat hearts had increased fibrosis over the left and right ventricles than did the control and lithium-treated REM sleep-deprived hearts.

### 2.3. Lithium Downregulates Profibrotic Signaling in Rats Deprived of REM Sleep

**Figure 3:**
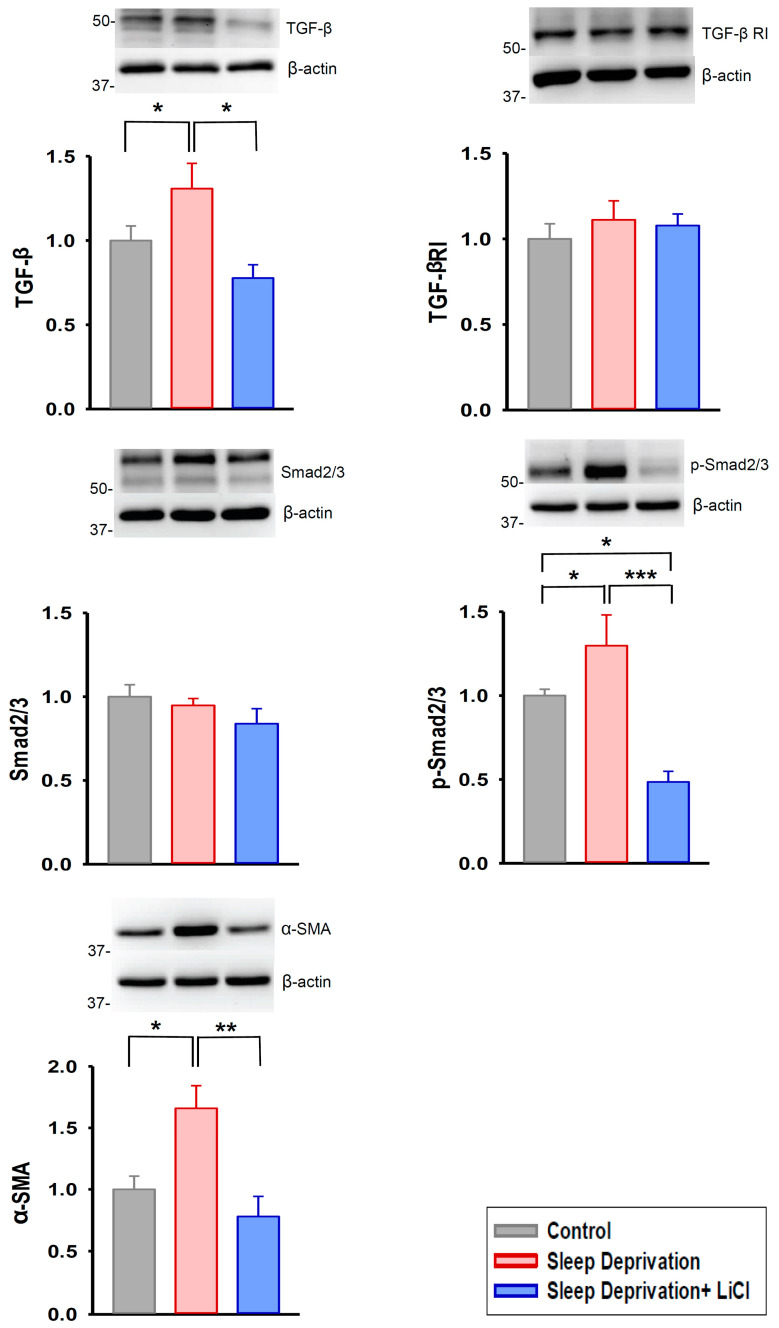
Transforming growth factor beta (TGF-β) signaling pathway in REM sleep deprivation with and without lithium treatment. The REM sleep-deprived hearts (*n* = 5) had greater expression levels of TGF-β, phosphorylated Smad 2/3, and α-smooth muscle actin (α-SMA) compared with the control (*n* = 5) and lithium-treated REM sleep-deprived (*n* = 5) hearts. * *p* < 0.05; ** *p* < 0.01; *** *p* < 0.001.

**Figure 4:**
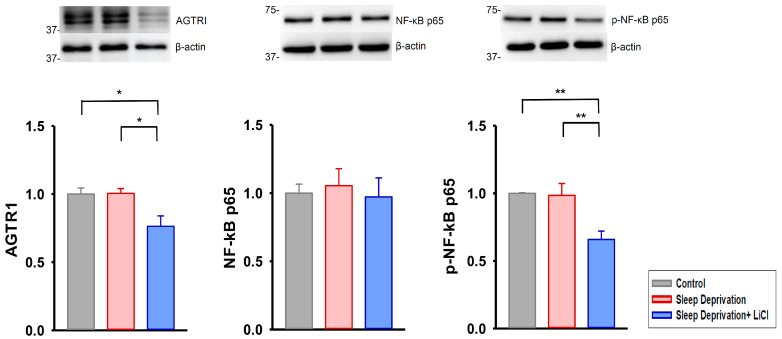
Angiotensin II cascade in REM sleep deprivation with and without lithium treatment. Compared with the REM sleep-deprived hearts (*n* = 5), the lithium-treated REM sleep-deprived hearts (*n* = 5) had lower expression levels of angiotensin II receptor type 1 (AGTR1) and phosphorylated nuclear factor kappa B (NF-κB) p65. * *p* < 0.05; ** *p* < 0.01.

**Figure 5:**
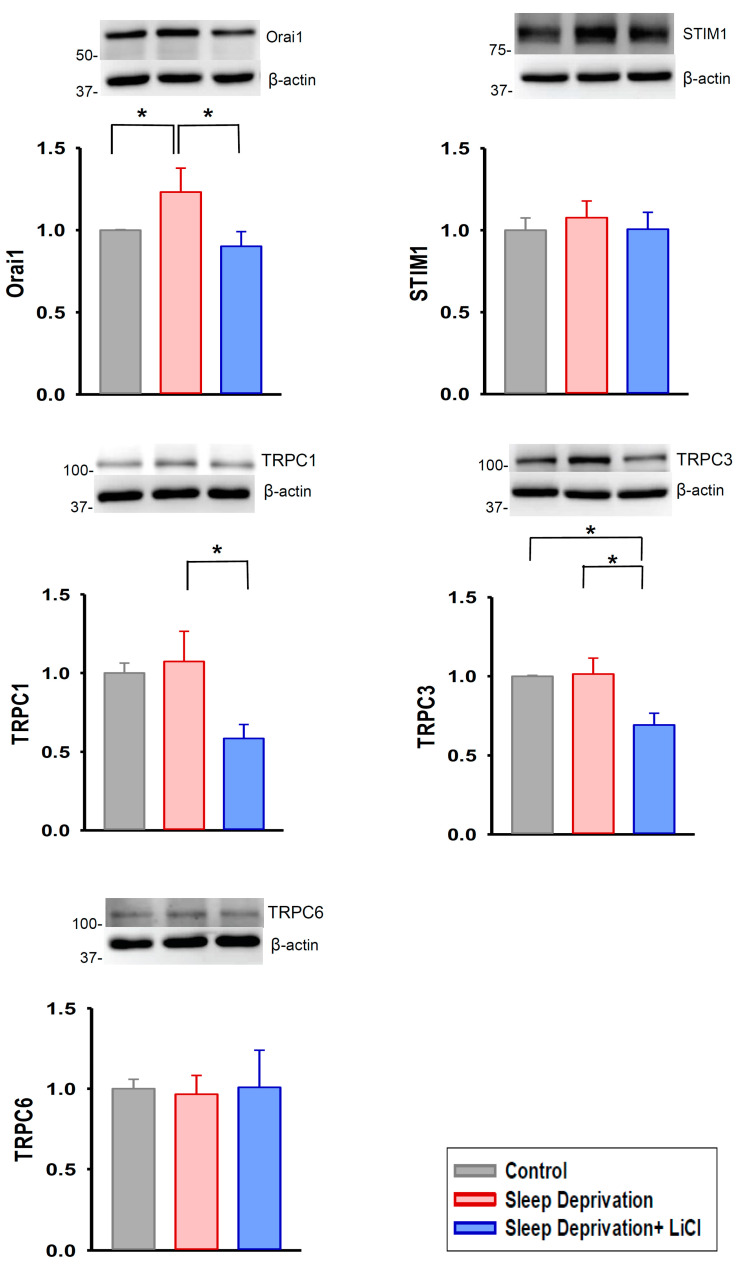
Expression levels of store-operated Ca^2+^ entry channels in REM sleep deprivation with and without lithium treatment. Compared with the REM sleep-deprived hearts (*n* = 5), the lithium-treated REM sleep-deprived hearts (*n* = 5) had lower expression levels of calcium release-activated calcium channel protein 1 (Orai1), transient receptor potential canonical (TRPC) 1 channel, and TRPC3 channel. * *p* < 0.05.

We further examined the effects of lithium treatment on profibrotic signaling in the REM sleep-deprived rat hearts; we discovered that these hearts had higher expression of transforming growth factor beta (TGF-β), phosphorylated Smad 2/3, and alpha-smooth muscle actin (α-SMA) than did the control and lithium-treated REM sleep-deprived hearts (Figure 3). Moreover, the lithium-treated REM sleep-deprived hearts had lower expression of angiotensin II receptor type 1 (AGTR1) and phosphorylated nuclear factor kappa B (NF-κB) p65 than did the hearts of the REM sleep-deprived rats (Figure 4). Because Ca^2+^ influx critically contributes to fibroblast activity [13,17], we investigated the expression levels of ion channels regulating store-operated Ca^2+^ entry and found that the lithium-treated REM sleep-deprived hearts had lower expression levels of calcium release-activated calcium channel protein 1 (Orai1), transient receptor potential canonical (TRPC) 1, and TRPC3 than did the REM sleep-deprived hearts (Figure 5). However, the lithium-treated REM sleep-deprived hearts had similar expression levels of TGF-β receptor type I (TGF-β RI), total Smad 2/3, total NF-κB p65, stromal interaction molecule 1 (STIM1), and TRPC6 to the REM sleep-deprived hearts.

## 3. Discussion

The present study demonstrated that REM sleep deprivation activated profibrotic signaling in the myocardium and induced cardiac fibrosis and dysfunction. The findings are consistent with those of previous studies [7,8,9], illustrating the development of cardiac fibrogenesis and ventricular dysfunction in rats deprived of REM sleep. This study suggests that lithium treatment may rescue cardiac remodeling in patients with bipolar disorder because the profibrotic signaling regulated by lithium in REM sleep-deprived hearts is also involved in the pathophysiology of bipolar disorder [18,19,20].

In agreement with the findings of our previous study [13], the present study found that lithium treatment downregulated the expression of Orai1 in myocardium of the REM sleep-deprived rats. This finding is consistent with previous clinical studies, indicating that lithium may reduce store-operated calcium entry in patients with bipolar disorder [21]. Previous research has shown that Orai1 is a critical ion channel in the regulation of store-operated Ca^2+^ entry in cardiac pathology [22]. Moreover, cardiac fibroblasts isolated from patients with heart failure possess an enhanced ability to synthesize collagen, which is linked to increases in store-operated Ca^2+^ entry and Orai1 expression [23]. These findings suggest that Orai1 is a crucial channel for the regulation of Ca^2+^ entry in cardiac fibrosis. Notably, a previous in vivo study has demonstrated that the inhibition of Orai1 prevents renal fibrosis [24]. Our present findings suggest that Orai1 inhibition has protective effects against fibrogenesis in REM sleep-deprived hearts.

Emerging evidence suggests that TRPCs are another class of ion channels that regulate store-operated Ca^2+^ entry and cardiac fibrogenesis [25,26]. In the TRPC family, the TRPC1, TRPC3, and TRPC6 channels are expressed in cardiac fibroblasts and play an essential role in the formation of cardiac fibrosis [26]. In particular, the present study found that in addition to the reduction of Orai1 expression, lithium treatment downregulated the TRPC1 and TRPC3 levels in REM sleep-deprived rat hearts. This finding is similar to those of other studies, suggesting that lithium treatment may decrease the expressions of the TRPC1 and TRPC3 genes in neurons or lymphoblasts of patients with bipolar disorder [27,28,29]. Given the evidence that TRPC channels may participate in store-operated Ca^2+^ entry dependent on Orai1 [30,31], future research needs to elucidate the specific roles of Orai1 and TRPC channels in the antifibrotic action of lithium in REM sleep-deprived hearts.

Among the predominant effectors promoting cardiac fibrosis, angiotensin II has been shown to activate NF-κB through AGTR1 [32,33,34]. Overexpression of the NF-κB subunits p50 and p65 further increases the transcripts of store-operated Ca^2+^ entry channels, including Orai1, TRPC1, and TRPC3 [35,36,37,38]. In the present study, we found that lithium treatment reduced the expression levels of AGTR1 and phosphorylated NF-κB p65 in the myocardium of REM sleep-deprived rats. This finding is in agreement with other findings in the literature, suggesting that lithium has the potential to regulate the angiotensin II and NF-κB pathways [39,40]. Together with our other main findings in this study, namely, that lithium treatment downregulated the expression levels of Orai1, TRPC1, and TRPC3 in the myocardium of REM sleep-deprived rats, our results suggest that the inhibition of angiotensin II and NF-κB signaling may contribute to the antifibrotic action of lithium in REM sleep-deprived hearts through the attenuation of store-operated Ca^2+^ entry.

**Figure 6:**
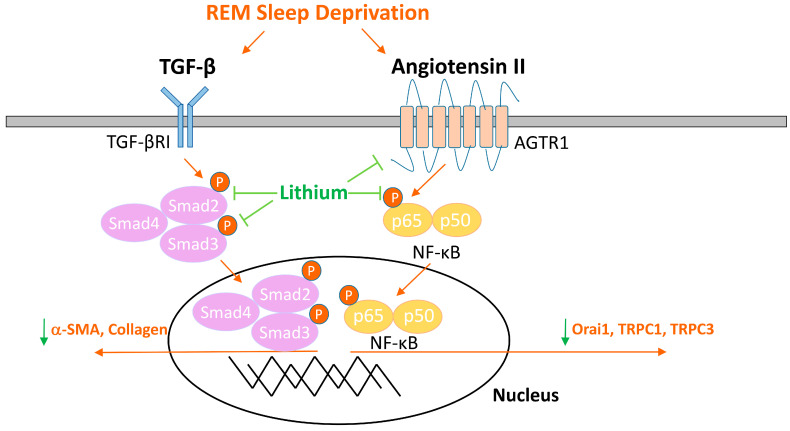
Schematic illustration of the proposed mechanisms for cardioprotection of lithium in REM sleep deprivation. Lithium may improve ventricular dysfunction and cardiac fibrosis in REM sleep-deprived hearts via the downregulation of the transforming growth factor beta signaling pathway, angiotensin II cascade, and store-operated Ca^2+^ entry. Abbreviations: α-SMA = α-smooth muscle actin; AGTR1 = angiotensin II receptor type 1; NF-κB = nuclear factor kappa B; Orai1 = calcium release-activated calcium channel protein 1; REM = rapid eye movement; TGF-β = transforming growth factor beta; TGF-β R1 = transforming growth factor beta receptor 1; TRPC1 *=* transient receptor potential canonical 1 channel; TRPC3 *=* transient receptor potential canonical 1 channel.

TGF-β is another crucial mediator of cardiac fibrogenesis [11,12]. Upon binding to the TGF-β receptor, TGF-β signaling induces the phosphorylation of Smad2 and Smad3 to form a complex with Smad4 [41]. Subsequently, the Smad2/3/4 complex translocates into the nucleus and activates the expression of profibrotic genes, including the myofibroblast differentiation marker α-SMA [41,42]. In the present study, we discovered that lithium treatment decreased the expression levels of TGF-β, phosphorylated Smad 2/3, and α-SMA in REM sleep-deprived hearts. The findings suggest that lithium treatment may alleviate cardiac fibrogenesis in individuals with REM sleep deprivation through its inhibitory effect on TGF-β signaling. Considering that angiotensin II also stimulates TGF-β released from fibroblasts [43,44], future mechanistic studies need to elucidate whether lithium inhibits TGF-β pathway in REM sleep-deprived hearts through the inhibition of angiotensin II signaling. As summarized in Figure 6, lithium treatment may improve ventricular contractibility and ameliorate cardiac fibrosis in REM sleep-deprived rats via blocking the activation of TGF-β and angiotensin II signaling and reducing store-operated Ca^2+^ entry.

When interpreting the data obtained in this study, several limitations need to be considered. First, previous studies have demonstrated that cardiac fibrogenesis is a complex process that involves a wide range of fibroblast-activating molecules [11,12]. The present study investigated associations between lithium treatment and well-known profibrotic pathways, including TGF-β, angiotensin II, and Ca^2+^ signaling; however, lithium may reduce cardiac fibrosis through additional signaling cascades. Second, we administered lithium from the first day of REM sleep deprivation; therefore, whether lithium can reverse pre-existing cardiac fibrosis remains unclear. Third, evidence from previous research suggests that the therapeutic effects of lithium are dose-dependent [45]. In the present study, lithium administered at a dosage of 1 mmol/kg per day produced plasma levels of 0.36 ± 0.03 mM. The concentrations were similar to those observed in previous studies demonstrating the antiarrhythmic and antihypertrophic effects of lithium in rat models [46,47]. To develop a greater understanding of lithium’s value in cardioprotection for clinical patients, additional studies are warranted to examine the dose-response effects of lithium on cardiac fibrosis.

In conclusion, the findings of this experimental study suggest that lithium treatment improves ventricular contractibility and attenuates cardiac fibrosis in REM sleep-deprived hearts. The downregulation of TGF-β, angiotensin II, and store-operated Ca^2+^ entry may contribute to these beneficial effects. To meaningfully translate our laboratory findings to patients, future trials are warranted to evaluate the cardioprotective effects of lithium by incorporating imaging biomarkers reflecting fibrogenic processes in the myocardium.

## 4. Materials and Methods

### 4.1. Animal Housing and Care

Male Wistar rats aged 8 weeks and weighing 250−300 g were used in this study. The rats were maintained on a 12:12-h light–dark cycle and were housed in pairs to ensure their social stability. Cage cleaning was performed by the staff at the Laboratory Animal Center of Taipei Medical University in accordance with standard procedures. Food and water were freely accessible to the rats. The study experimental protocol was approved by the Institutional Animal Care and Use Committee of Taipei Medical University (LAC−2020−0342).

### 4.2. REM Sleep Deprivation Procedure and Lithium Treatment

**Figure 7:**
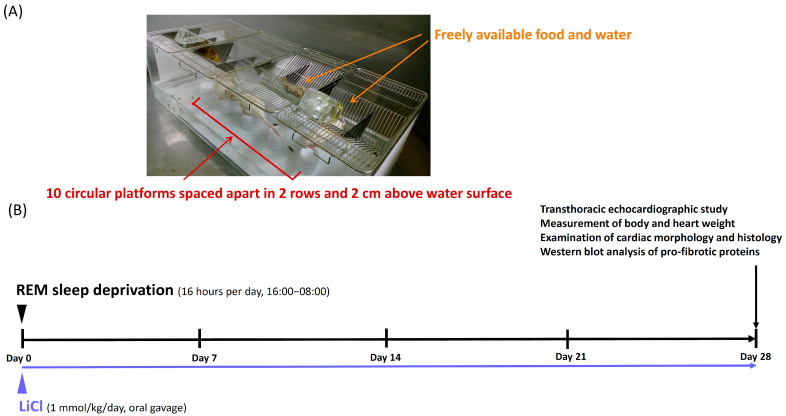
Experimental design of this study. (**A**) Illustration of water tank used for induction of REM sleep deprivation in male Wistar rats. (**B**) Illustration summarizes the experimental protocol. The rats in the REM sleep deprivation group were subjected to REM sleep deprivation for 16 h per day (16:00−08:00) over 4 weeks. For the group with lithium treatment, LiCl at a dosage of 1 mmol/kg was administered by oral gavage once a day for 4 weeks.

Each rat was randomly assigned to one of the following three groups: (a) control, (b) REM sleep deprivation without lithium treatment, and (c) REM sleep deprivation with lithium treatment. The REM sleep deprivation procedure was performed using the modified multiple platform method, which consisted of placing the rats inside a water tank of the following dimensions: 100 cm (length) × 45 cm (width) × 30 cm (height) (Figure 7A). The water tank contained 10 circular platforms positioned in two rows. Each platform was 6 cm in diameter, separated from the other platforms in its row by 8 cm on each side, 10-cm high, and fixed to the bottom of the tank. Throughout the study period, food and drinking water were freely available to the rats. The water level in the tank was maintained at 2 cm below the platforms, and this water was changed daily. This experimental design enabled the rats to move around inside the tank by jumping from one platform to another during their waking hours. The idea underlying the experimental design was that the rats would fall off their platforms during REM sleep because of muscle atonia and would wake up upon contact with the water, resulting in the deprivation of REM sleep. The rats in the REM sleep deprivation group were subjected to such deprivation for 16 h per day (16:00−08:00) over 4 weeks. For the remaining 8 h (8:00−16:00), the rats were transferred back to their home cages. For the group with lithium treatment, LiCl (#62476; Sigma-Aldrich, St. Louis, MO, USA) at a dosage of 1 mmol/kg was administered by oral gavage once a day for 4 weeks (Figure 7B). This dosage was chosen based on the literature [46,47].

### 4.3. Transthoracic Echocardiographic Examination

A transthoracic echocardiographic study was conducted in the rats when the rats were aged 12 weeks in accordance with our previously described method [48,49,50]. In brief, transthoracic echocardiography was conducted using the Vivid I ultrasound cardiovascular system (GEHealthcare, Haifa, Israel) under isoflurane anesthesia (5% for induction and 2% for maintenance). M-Mode tracing of the left ventricle was used to measure interventricular septal thickness at end-diastole, LVPWd, internal diameter of the left ventricle at end-diastole, LVIDs, FS, and EF.

### 4.4. Histological Study

The rats were anesthetized by deep inhalation of 5% isoflurane and were euthanized at 12 weeks of age. After euthanasia, their hearts were rapidly excised, weighed, formalin-fixed, and paraffin embedded. Subsequently, the paraffin-embedded hearts were serially sectioned at 5 μm intervals. The slices were then stained with hematoxylin–eosin (HE) and Masson’s trichrome stains. Masson’s trichrome is a three-color staining assay used to identify the collagen composition in tissue. Collagen fibers were stained blue, and the cytosol was stained red. After staining, the collagen volume fraction (the ratio of the total collagen surface area to the surface area of each individual chamber) was computed to assess fibrosis severity using HistoQuest Analysis Software (version 4.0; TissueGnostics, Vienna, Austria) in accordance with our previously described method [49].

### 4.5. Western Blotting

Freshly isolated ventricular tissue samples were rinsed in cold saline solution and frozen in liquid nitrogen at −80 °C until protein isolation. Equal amounts of protein were then resolved by sodium dodecyl sulfate–polyacrylamide gel electrophoresis, followed by the electrophoretic transfer of proteins onto nitrocellulose membranes. Blots were probed with the following antibodies against regulator proteins involved in cardiac fibrogenesis: TGF-β (1:2000, polyclonal, #3711; Cell Signaling Technology, Beverly, MA, USA), TGF-β RI (1:1000, polyclonal, #sc-398; Santa Cruz Biotechnology, Santa Cruz, CA, USA), total Smad 2/3 (1:2000, monoclonal, #8685; Cell Signaling Technology), phosphorylated Smad 2/3 (1:2000, monoclonal, #8828; Cell Signaling Technology), α-SMA (1:5000, monoclonal, #ab7817; Abcam, Cambridge, UK), AGTR1 (1:2000, polyclonal, #AAR-011; Alomone Labs, Jerusalem, Israel), total NF-κB p65 (1:2000, monoclonal, #8242; Cell Signaling Technology), phosphorylated NF-κB p65 (1:2000, monoclonal, #3033; Cell Signaling Technology), Orai1 (1:2000, polyclonal, #4281; ProSci Incorporated, Poway, CA, USA), STIM1 (1:2000, monoclonal, #610954; BD Transduction Laboratories, San Diego, CA, USA), TRPC 1 channel (1:2000, polyclonal, #ACC-010; Alomone Labs), TRPC3 (1:5000, polyclonal, #ACC-016; Alomone Labs), and TRPC6 (1:2000, polyclonal, #ACC-017; Alomone Labs). Bound antibodies were detected using an enhanced chemiluminescence detection system (Millipore, Darmstadt, Germany) and analyzed with AlphaEaseFC software (Alpha Innotech, San Leandro, CA, USA). Targeted bands were normalized to beta (β)-actin (1:10,000, polyclonal, #ab6274; Abcam) to confirm equal protein loading.

### 4.6. Statistical Analysis

The quantitative data are expressed as the mean ± the standard error of the mean. A repeated measures or nonrepeated measures one-way analysis of variance with a Tukey’s posthoc test was used to compare the three groups. The Kruskal–Wallis test was applied when the normality assumption was not met. *p* < 0.05 was considered statistically significant.

## Data Availability

The data presented in this study are available on request from the corresponding author.
